# Supplementary material for: Effectiveness and Safety of Ayurvedic Medicines in Type 2 Diabetes Mellitus Management: A Systematic Review and Meta-Analysis
Source: Front Pharmacol. 2022 Jun 8;13:821810. doi: 10.3389/fphar.2022.821810 (PMC9213670; doi:10.3389/fphar.2022.821810)
Supplement: Supplementary file 1 [file DataSheet1.zip › Supplementary Material/Forest and Funnel Plots/Zingiber officinale/Fasting insulin.pdf]

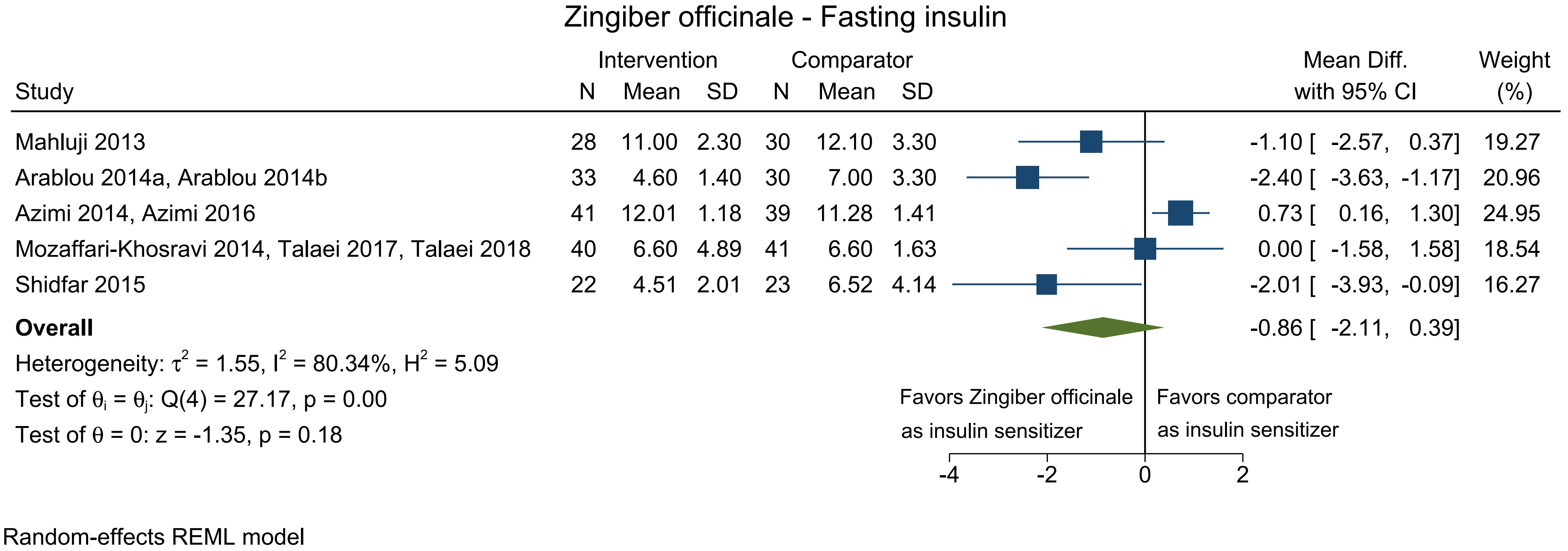
Zingiber officinale - Fasting insulin
Intervention
Comparator
Study
N
Mean
SD
N
Mean
SD
Mean Diff. with 95% CI
Weight (%)
Mahluji 2013 28 11.00 2.30 30 12.10 3.30 -1.10 [-2.57, 0.37] 19.27
Arablou 2014a, Arablou 2014b 33 4.60 1.40 30 7.00 3.30 -2.40 [-3.63, -1.17] 20.96
Azimi 2014, Azimi 2016 41 12.01 1.18 39 11.28 1.41 0.73 [0.16, 1.30] 24.95
Mozaffari-Khosravi 2014, Talaei 2017, Talaei 2018 40 6.60 4.89 41 6.60 1.63 0.00 [-1.58, 1.58] 18.54
Shidfar 2015 22 4.51 2.01 23 6.52 4.14 -2.01 [-3.93, -0.09] 16.27
Overall -0.86 [-2.11, 0.39]
Heterogeneity: $\tau^2 = 1.55$, $I^2 = 80.34\%$, $H^2 = 5.09$
Test of $\theta_i = \theta_j$: $Q(4) = 27.17$, $p = 0.00$
Test of $\theta = 0$: $z = -1.35$, $p = 0.18$
Favors Zingiber officinale as insulin sensitizer
Favors comparator as insulin sensitizer
-4
-2
0
2
Random-effects REML model
